# Supplementary material for: Arsenic‐Containing Phosphatidylcholines: A New Group of Arsenolipids Discovered in Herring Caviar
Source: Angew Chem Int Ed Engl. 2016 Mar 21;55(17):5259–62. doi: 10.1002/anie.201512031 (PMC4950057; doi:10.1002/anie.201512031)
Supplement: Supplementary file 1 — Supplementary [file ANIE-55-5259-s001.pdf]

Angewandte Chemie
Eine Zeitschrift der Gesellschaft Deutscher Chemiker

Supporting Information

Arsenic-containing Phosphatidylcholines: a New Group of Arsenolipids Discovered in Herring Caviar

Sandra A. Viczek, Kenneth B. Jensen, and Kevin A. Francesconi*

anie_201512031_sm_miscellaneous_information.pdf

Supporting Information

Contents

Experimental S2

Chemicals and standards. S2

Instrumentation. S2

Extraction of arsenolipids. S2

Total arsenic content. S2

Screening for arsenolipids by RP-HPLC/ICPMS S3

Identification of arsenolipids by high resolution ESIMS. S3

Further results, tables and figures S4

Arsenolipids in herring caviar. S4

MS/MS spectrum of AsFA 528 .... S6

MS/MS spectra of arsenic-containing phosphatidylcholines S7

Simulated spectra of arsenic-containing phosphatidylcholines S10

MS/MS spectra of the arsenic-containing phosphatidylethanolamine S15

Simulated spectra of the arsenic-containing phosphatidylethanolamine S16

References S17

Experimental

Chemicals and standards. Water (resistivity: 18.2 MΩ cm) was obtained from a Milli-Q system (Millipore GmbH, Vienna, Austria), Dichloromethane 99.8% (HiPerSolv CHROMANORM, VWR Chemicals, Radnor, United States), Methanol 99.9% (HiPerSolv CHROMANORM, VWR Chemicals, Radnor, United states), Ethanol abs. 100% (Chem-Lab NV, Zedelgem, Belgium), Formic acid 98% (Carl Roth GmbH + Co. KG, Karlsruhe, Germany), Nitric acid 65% (Carl Roth GmbH + Co. KG, Karlsruhe, Germany) subboiled in house (duoPUR, MLS GmbH, Leutkirch, Germany).
Standard compounds of AsHC332, AsHC360, AsHC 444, AsFA 362, AsFA 388, and AsFA 418 were synthesized in-house according to Taleshi et al.[1] and prepared by dissolving 7.5 ± 0.2 µg (as As) in ethanol (1 mL)

Instrumentation. Samples were freeze-dried by a lyophilizer (Christ gamma 1-15 LSC freeze-drier, Martin Christ Gefriertrocknungsanlagen GmbH, Osterode am Harz, Germany), acid digested with an Ultraclave IV microwave system (MLS GmbH, Leutkirch, Germany) or extracted with an extractor (Stuart tube rotator SB2, Bibby Scientific Limited, Staffordshire, United Kingdom) constantly rotating at 20 rpm. Solvents were evaporated by a centrifugal lyophilizer (Christ RVC 2-33 CD plus, Martin Christ Gefriertrocknungsanlagen GmbH, Osterode am Harz, Germany). Total Arsenic contents were determined on an Agilent 7900 ICPMS. HPLC/ICPMS measurements were carried out on an Agilent 1100 series HPLC system connected an Agilent 7500ce series ICPMS (Agilent Technologies, Waldbronn, Germany)equipped with an ESI PC3 Peltier cooled cyclonic spray chamber (Elemental Scientific, Omaha, USA) and an Ari Mist HP nebulizer (Burgerner, Mississauga, Canada). A Dionex Ultimate 3000 series HPLC connected to a Q-Exactive Hybrid Quadrupole-Orbitrap MS (Thermo Fischer Scientific, Erlangen, Germany) was used for high resolution-ESIMS measurements.

Extraction of arsenolipids. Freeze-dried herring caviar (≈150 mg, weighed to 0.1 mg) was extracted twice with DCM/MeOH 2:1 v/v (4 mL) on an extractor for 2 hours. The supernatants were removed after centrifugation (15 min, 2133 G), combined, and water was added to the combined supernatants to reach a DCM/MeOH/water ratio of 2:1:1. The mixture was extracted for another 2 hours before centrifuging (15 min, 2133 G) to separate the layers. The organic layer was evaporated to dryness (10 mbar), re-dissolved in ethanol (700 µL) by vortexing and ultrasonication, filtered (syringe filters, nylon, 0.2 µm pore size, Markus Bruckner Analysentechnik, Linz, Austria) and measured without further cleanup. All steps were carried out at room temperature and in case of HRMS measurements glassware was used.

Total arsenic content. Samples of freeze dried material (200 mg weighed to 0.1 mg) or defined amounts extracts and pellets were weighed into quartz tubes and 3 mL of water and 2 mL of HNO_3 were added (extracts were first evaporated to dryness in a drying oven at 80°C). The tubes were covered with Teflon caps and placed in a Teflon rack. Together with each set of samples, a set of digestion blanks (3 mL water and 2 mL HNO_3, n=3) and at least one set of certified reference material (DOLT-3 (codfish liver), TORT-2 (lobster hepatopancreas) or IAEA 407 (fish tissue); digest of 50 mg in 3 mL water and 2 mL HNO_3, n=3) was placed in the system. An absorbance solution (300 g of water and 5 g of concentrated H_2SO_4) was prepared in the instrument's vessel. The vessel and tubes were transferred to the microwave system which was then filled with argon to reach a pressure of 40 bar. A temperature program as depicted in Figure S1 was applied.

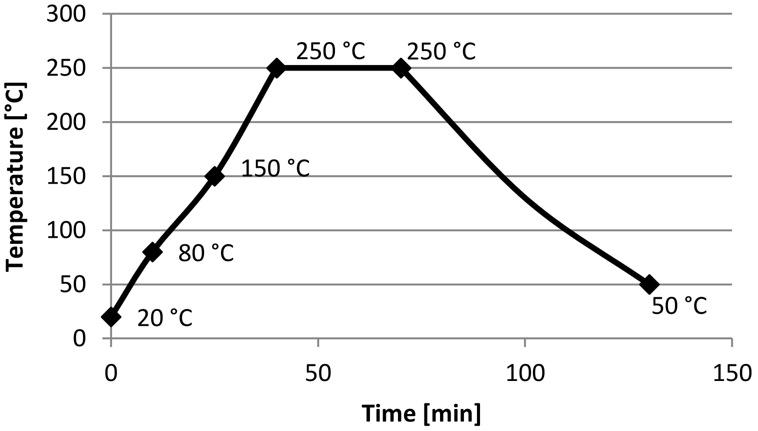

Time [min]	Temperature [°C]
0	20
10	80
25	150
40	250
70	250
130	50

Figure S1. Temperature program for microwave-assisted acid digestion with UltraCLAVE IV

Clear, colorless digest solutions were obtained and allowed to cool before quantitatively transferring them to 15 mL polypropylene tubes. 1 mL of an internal standard solution containing 100 µg/L of Ge, In and Te was added to each sample. The samples were filled with water to reach a total volume of 10 mL and an internal standard concentration of 10 µg/L.

Screening for arsenolipids by RP-HPLC/ICPMS. Separation was achieved by reversed-phase HPLC (Shodex Asahipak ODP-50 4D C18 column (4.6 x 150 mm) with guard column ODP-50G 4A (4.6 x 10 mm), particle size 5 µm (Showa Denko Europe GmbH, Munich, Germany), injection volume 50 µL) and a gradient elution with water containing 0.1% formic acid and EtOH containing 0.1% formic acid: 0-1 min 30% EtOH, 1-33 min 30-100% EtOH, 33-35 min 100% EtOH, 35-45 min 30% EtOH at a constant flow rate of 0.5 mL/min. A passive splitter (Analytical Scientific Instruments, Richmond, USA) was used to direct 10% of the effluent to the ICPMS and 90% to waste. A support flow (1% formic acid and 10 µg L^{-1} In, Ge, and Te in water, flow rate 0.5 mL) introduced by a t-piece after the splitter was used to dilute the HPLC effluent. Gradient compensation was carried out with 10% EtOH in water constantly introduced through the makeup gas inlet with the ISIS pump (0.02 ppm). Signals at m/z 75 (^{75}As and $^{40}Ar^{35}Cl$ interference) were recorded at integration times of 0.3 s, and at integration times of 0.05 s for m/z 77 (^{77}Se or $^{40}Ar^{37}Cl$ to account for chlorine interferences), m/z 53 (^{53}Cr or $^{40}Ar^{13}C$ to monitor the carbon content), m/z 72 (^{72}Ge), m/z 74 (^{74}Ge), m/z 115 (^{115}Te), and m/z 125 (^{125}In).

Identification of arsenolipids by high resolution ESIMS. Chromatographic conditions were the same as for ICPMS measurements, the injection volume was reduced to 20 µL. Samples were measured in positive mode with nitrogen as the drying gas, a capillary voltage of 3500 V, and a capillary temperature of 320°C. Data dependent MS/MS mode was used with the following settings: Full scan at a resolution of 70,000 between m/z 300-1100 Thomson with a MaxInjectionTime of 100 msec, and for the data-dependent MS/MS part: Isolation window 0.4 Thomson, Resolution: 17500, AutomaticGainControl: 10^5, MaxIT: 50 msec, loop count: 5, intensity threshold: $2*10^4$, so called NormalizedCollisionEnergy: 30 and 50, Dynamic exclusion time: 4 sec and also excluding ^{13}C-isotopes. Typically 9,000-10,000 MS/MS spectra of about 700 distinct ions from the full scan region were obtained.

Further results, tables and figures

Arsenolipids in herring caviar. Table S1 gives details on the caviar samples (if available), while table S2 gives an overview of all arsenolipids identified in three samples of herring caviar. Figure S2 gives structures for all found arsenolipids for which no structure was given in the communication itself.

Table S1. Origin of samples

Fish roe sample	Species	Catch area of fish	Location of producer
Herring A, black	*Clupea harengus*	Northeast Atlantic (Norwegian Sea), FAO 27	Germany
Herring B, black	*Clupea harengus*	-	Sweden
Herring C, red	*Clupea harengus*	-	Sweden
Chinook Salmon	*Oncorhynchus tshawytscha*	Alaska	Denmark

Table S2. Exact masses and Δm/m values for samples of herring roe

Compound abbreviation	Formula (neutral species)	$[M+H]^+$ calculated	Herring A		Herring B		Herring C	
			$[M+H]^+$ measured	|Δm/m| [ppm]	$[M+H]^+$ measured	|Δm/m| [ppm]	$[M+H]^+$ measured	|Δm/m| [ppm]
**AsFA 334**	$C_{15}H_{31}O_3As$	335.1562	335.1564	0.71	335.1560	0.46	335.1564	0.71
**AsFA 362**	$C_{17}H_{35}O_3As$	363.1875	363.1877	0.43	363.1875	0.10	363.1874	0.15
**AsFA 388**	$C_{19}H_{37}O_3As$	389.2031	389.2035	0.81	389.2033	0.43		
**AsFA 390**	$C_{19}H_{39}O_3As$	391.2188	391.2192	0.99	391.2197	2.24	391.2195	1.83
**AsFA 436**	$C_{23}H_{37}O_3As$	437.2031	437.2032	0.18	437.2029	0.60	437.2030	0.26
**AsFA 448**	$C_{24}H_{37}O_3As$	449.2031	449.2030	0.38	449.2029	0.52	391.2195	0.18
**AsFA 528**	$C_{30}H_{45}O_3As$	529.2657	529.2654	0.69	529.2654	0.57	529.2654	0.57
**AsHC 330**	$C_{17}H_{35}OAs$	331.1977	331.1976	0.28			331.1973	1.01
**AsHC 332**	$C_{17}H_{37}OAs$	333.2130	333.2131	0.64	333.2132	0.25	333.2133	0.16
**AsHC 346**	$C_{18}H_{39}OAs$	347.2290					347.2282	2.26
**AsHC 358**	$C_{19}H_{39}OAs$	359.2290	359.2291	0.46				
**AsHC 360**	$C_{19}H_{41}OAs$	361.2446	361.2445	0.29	361.2445	0.29	361.2444	0.54
**AsHC 404**	$C_{23}H_{37}OAs$	405.2133	405.2132	0.21	405.2134	0.24	405.2134	<0.10
**AsPC 885**	$C_{45}H_{81}O_9NPAs$	886.4937	886.4950	1.39	886.4945	0.84	886.4918	2.26
**AsPC 911**	$C_{47}H_{83}O_9NPAs$	912.5094	912.5103	0.91	912.5085	0.96	912.5094	<0.10
**AsPC 939**	$C_{49}H_{87}O_9NPAs$	940.5407	940.5397	1.05	940.5411	0.45	940.5444	3.88
**AsPC 985**	$C_{53}H_{85}O_9NPAs$	986.5250	986.5248	0.23	986.5241	1.03	986.5242	0.91
**AsPC 997**	$C_{54}H_{85}O_9NPAs$	998.5250	998.5253	0.20	998.5245	0.53	998.5243	0.78

AsFA 334

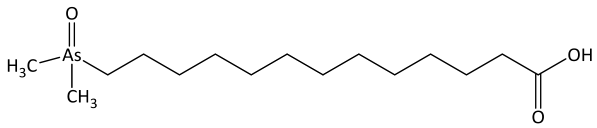

AsFA 362

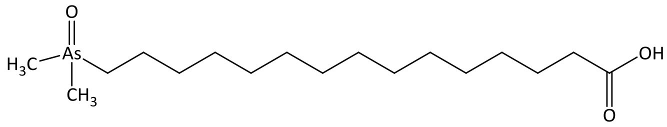

AsFA 388

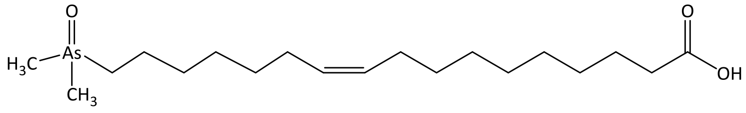

AsFA 390

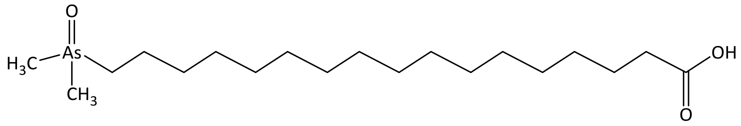

AsFA 436

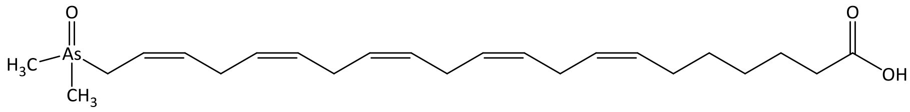

AsFA 448

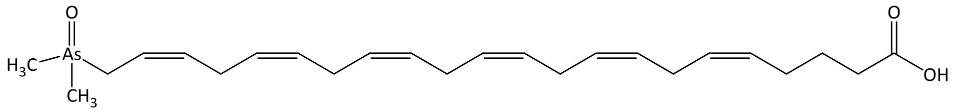

AsHC 330

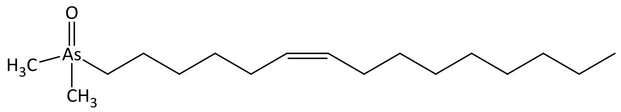

AsHC 332

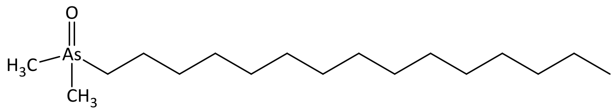

AsHC 404

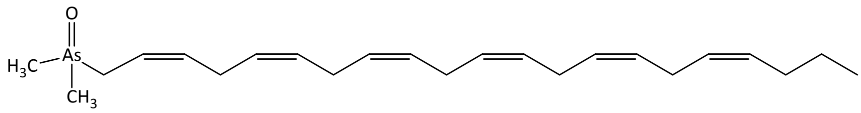

AsHC 346

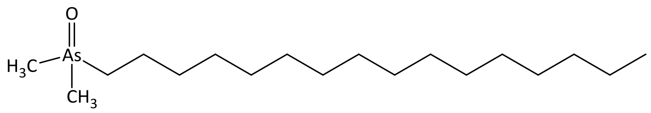

AsHC 358

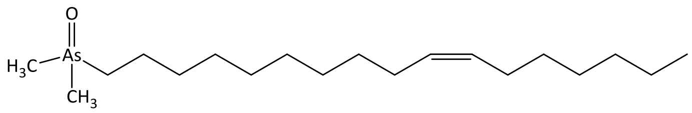

AsHC 360

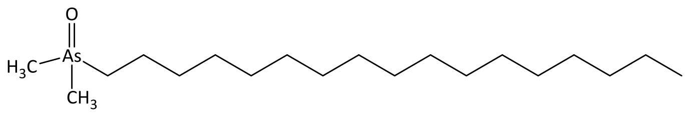

Figure S2. Structures of known arsenolipids found in herring roe samples. Position and geometry of double bonds have not been determined and were assigned by analogy to commonly occurring non-arsenic lipids.

MS/MS spectrum of AsFA 528

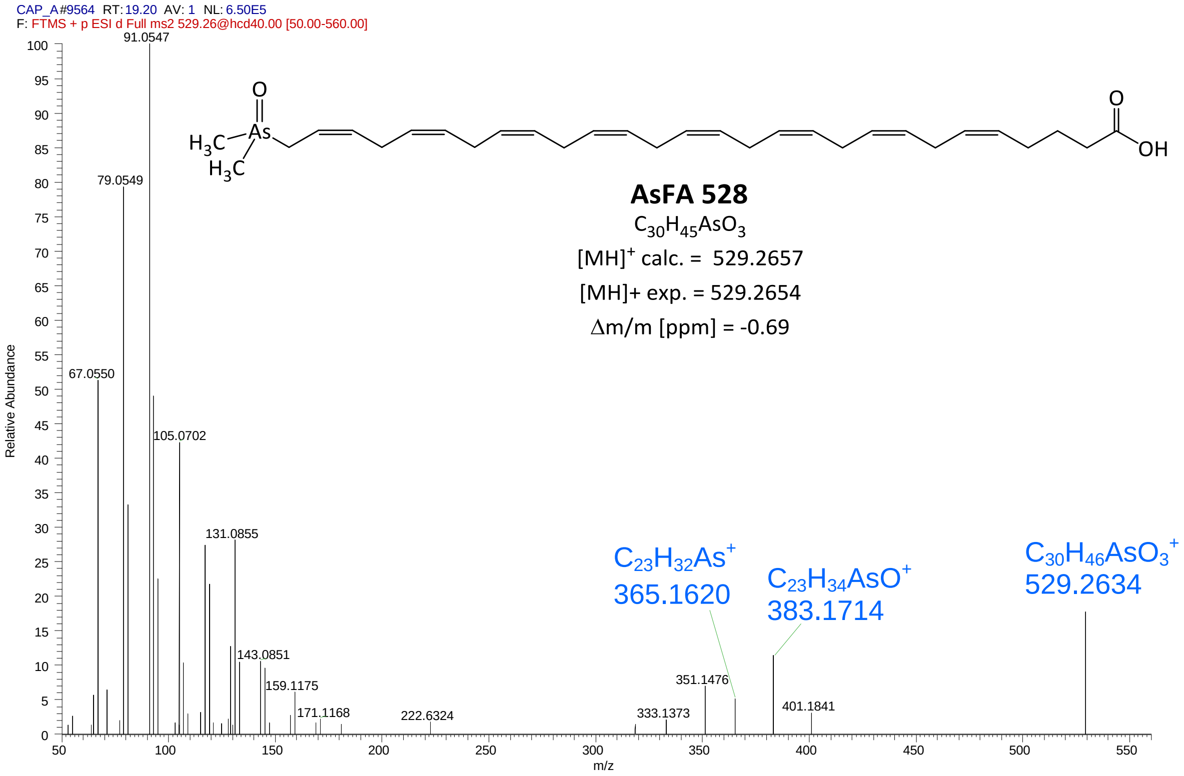

Figure S3. MS/MS spectrum and proposed structure of AsFA 528. Position and geometry of double bonds have not been determined.

MS/MS spectra of arsenic-containing phosphatidylcholines

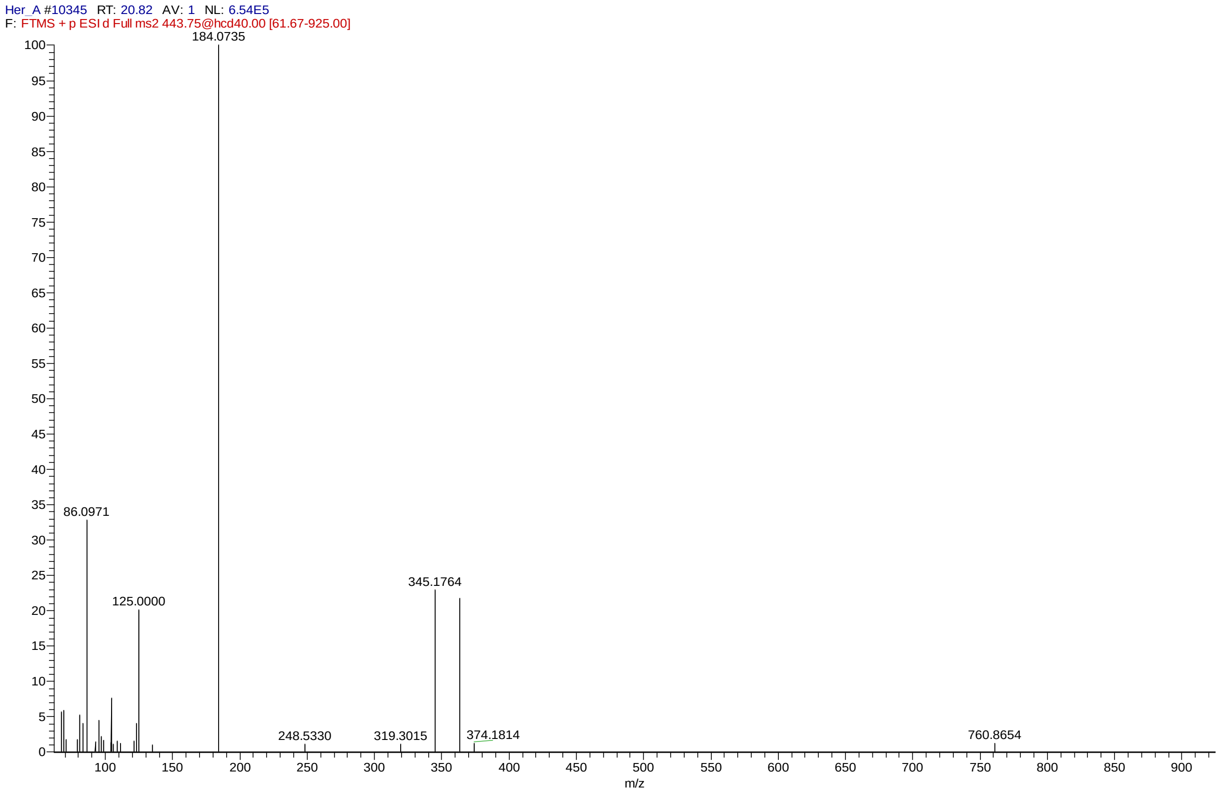

Figure S4. MS/MS spectrum of the doubly charged species of AsPC 885 at m/z=443.75 (neutral: $C_{45}H_{81}O_9NAsP$)

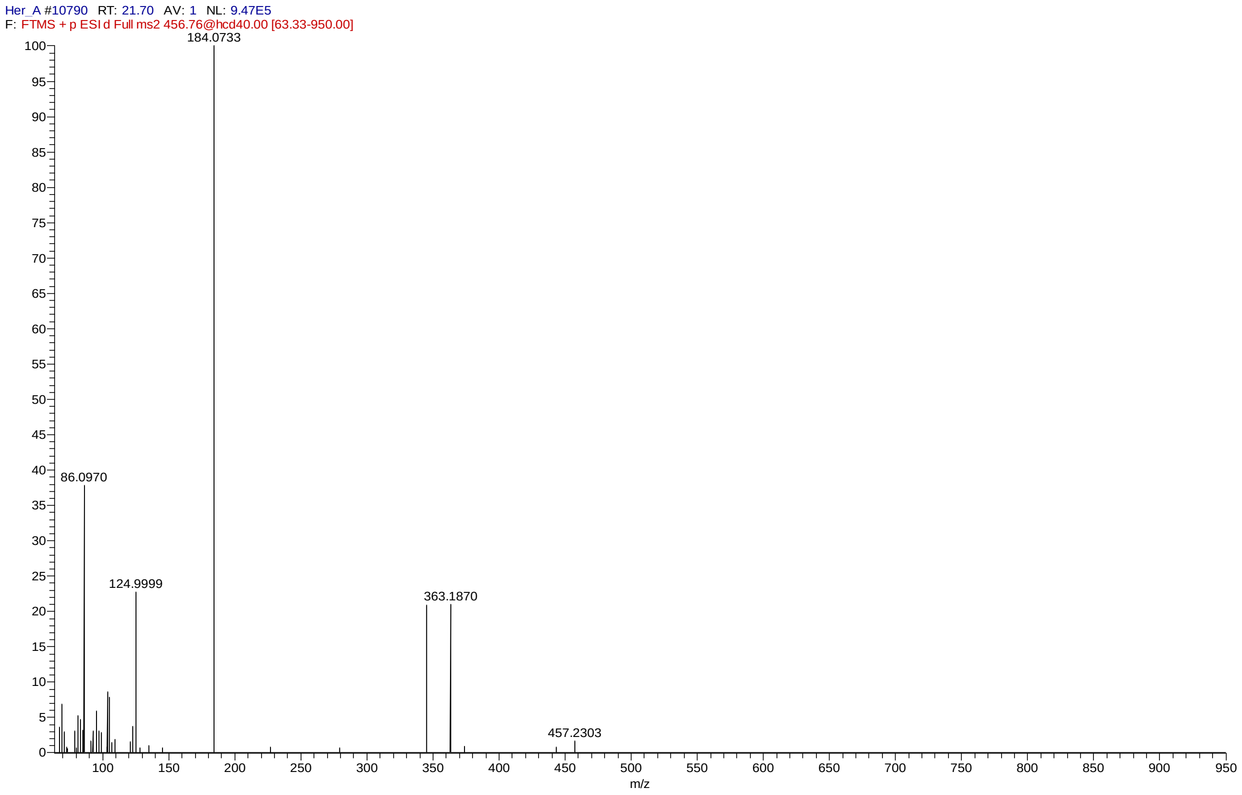

Figure S5. MS/MS spectrum of the doubly charged species of AsPC 911 at m/z=456.76. (neutral: $C_{47}H_{83}O_9NAsP$)

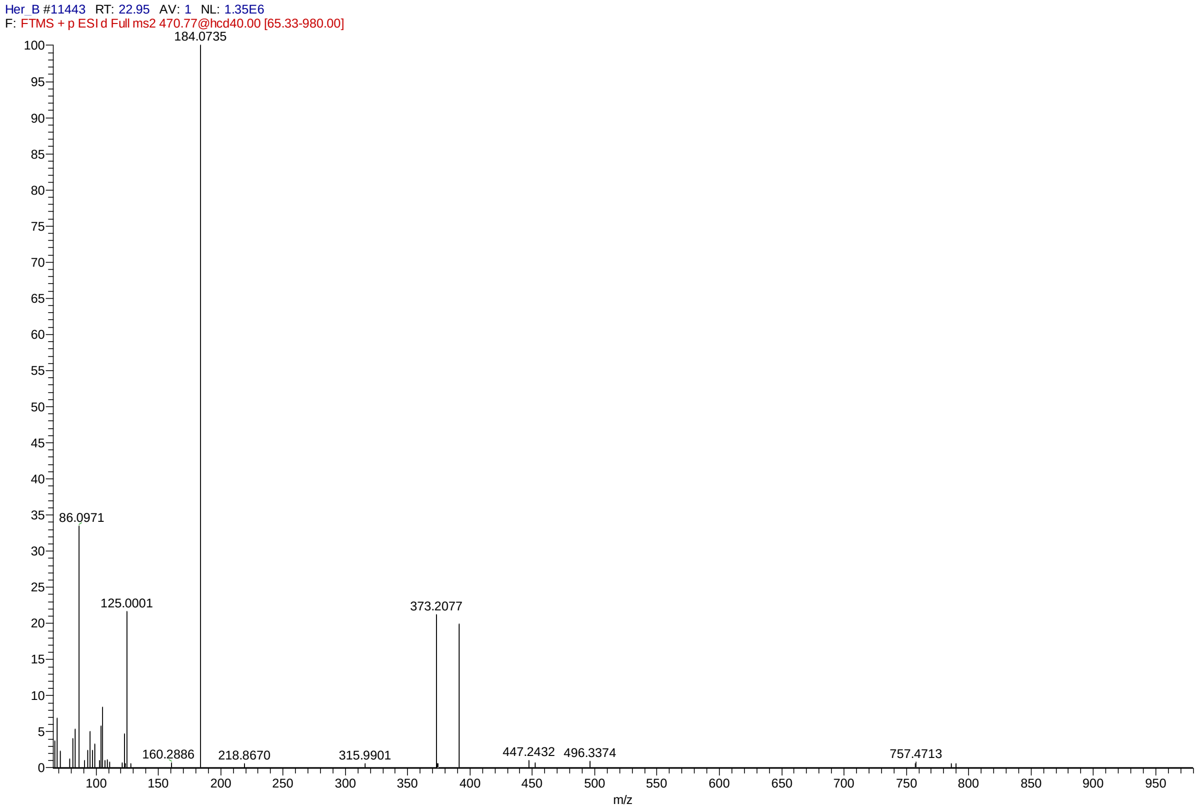

Figure S6. MS/MS spectrum of the doubly charged species of AsPC 939 at m/z=470.77. (neutral: $C_{49}H_{87}O_9NAsP$)

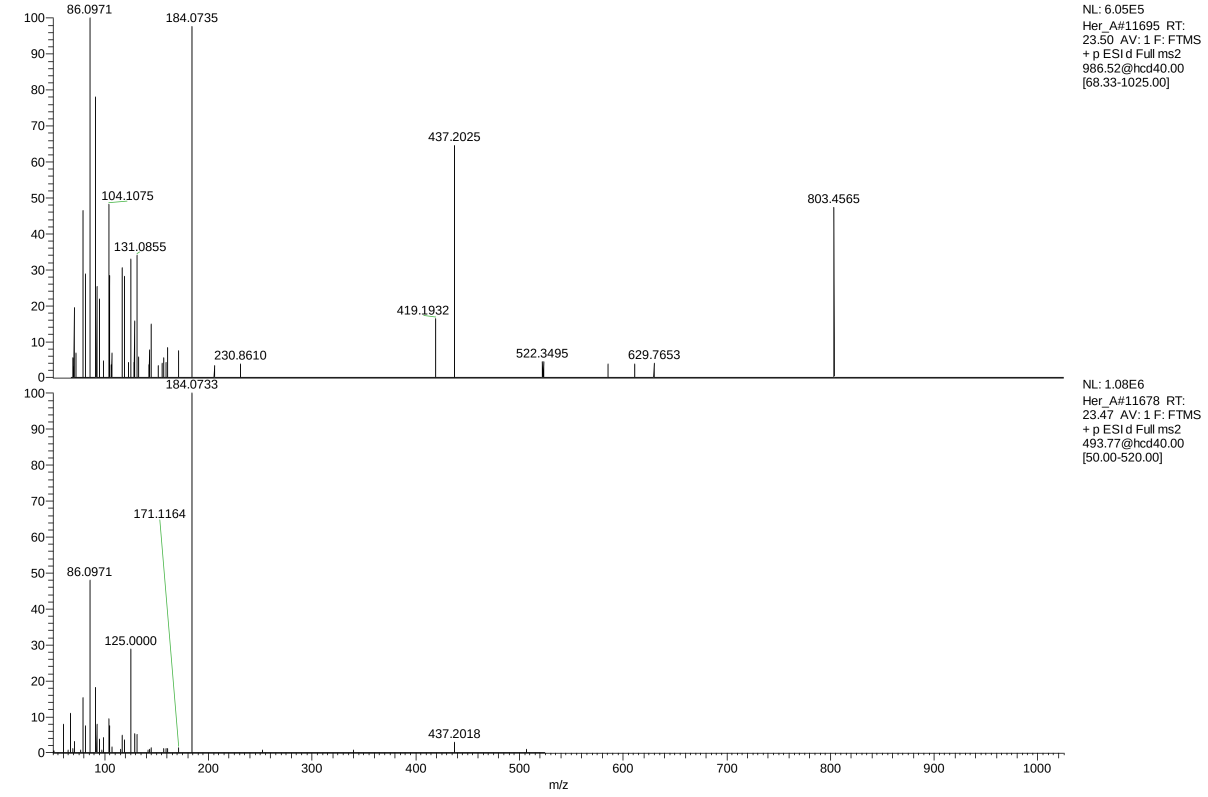

Figure S7. MS/MS spectrum of the singly charged (above, m/z=986.52) and doubly charged (below, m/z=493.77) species of AsPC 985. (neutral: $C_{53}H_{85}O_9NAsP$)

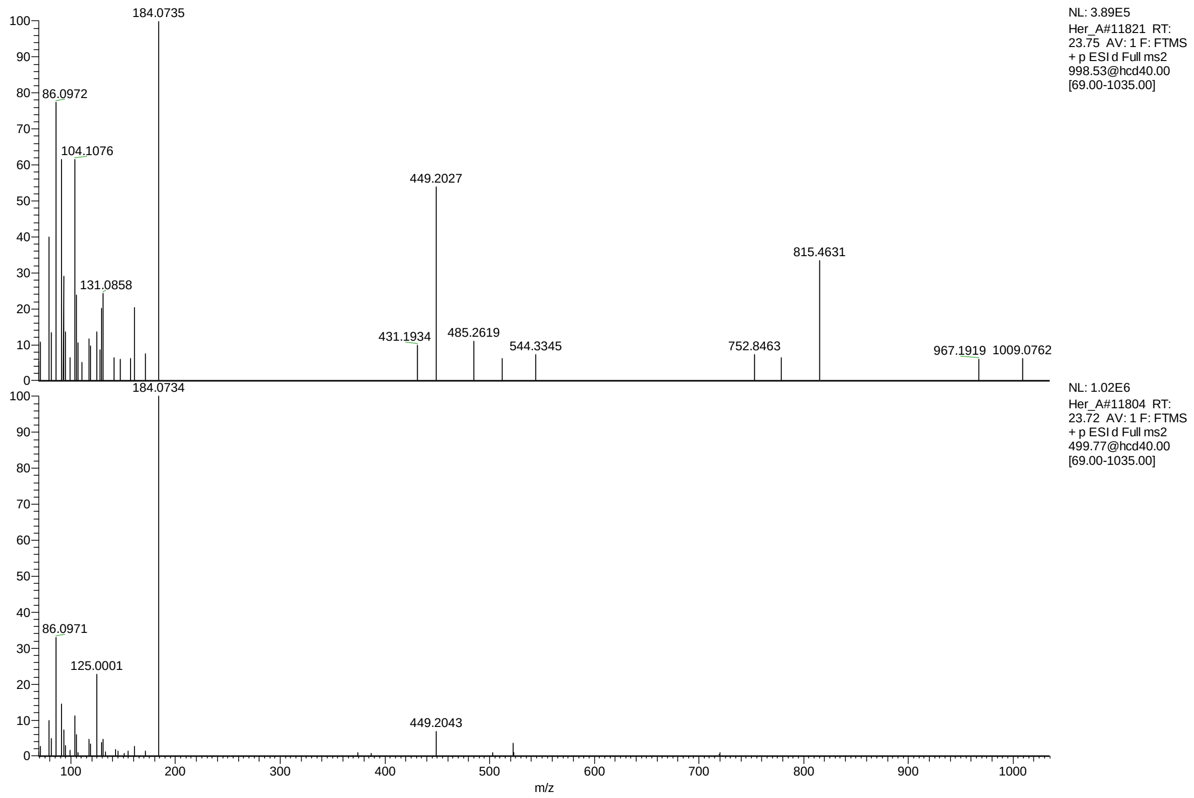

Figure S8. MS/MS spectrum of the singly charged (above, m/z=998.53) and doubly charged (below, m/z=499.77) species of AsPC 997. (neutral: $C_{54}H_{85}O_9NAsP$)

Simulated spectra of arsenic-containing phosphatidylcholines

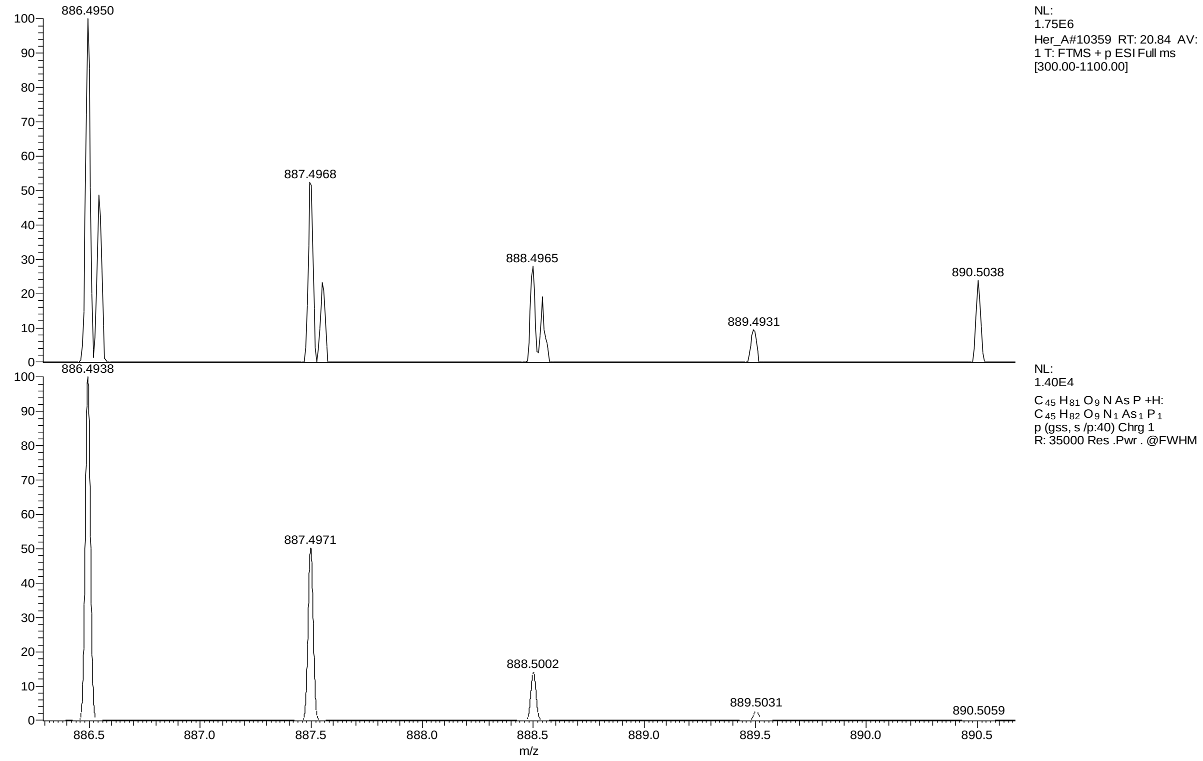

Figure S9. Measured (above) and simulated (below) isotopic pattern of AsPC 885 (singly charged, $C_{45}H_{81}O_9NAsP\_H^+$)

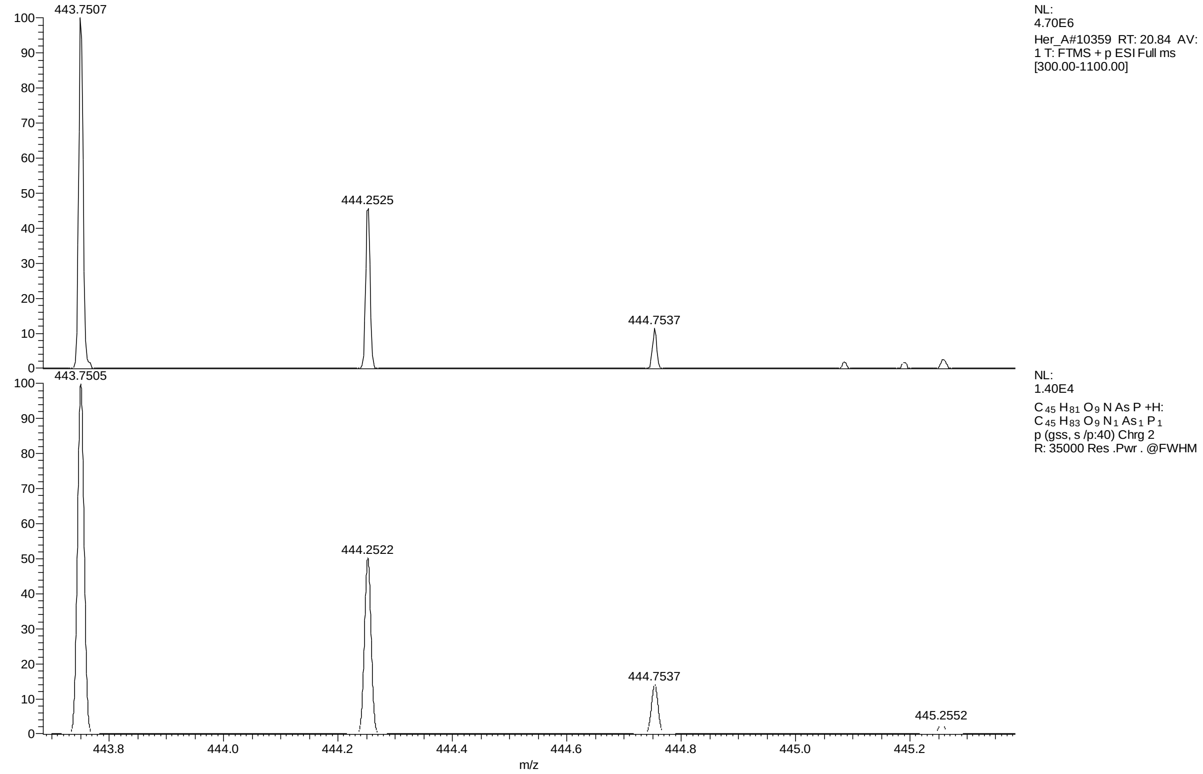

Figure S10. Measured (above) and simulated (below) isotopic pattern of AsPC 885 (doubly charged, $C_{45}H_{81}O_9NAsP\_2H^+$)

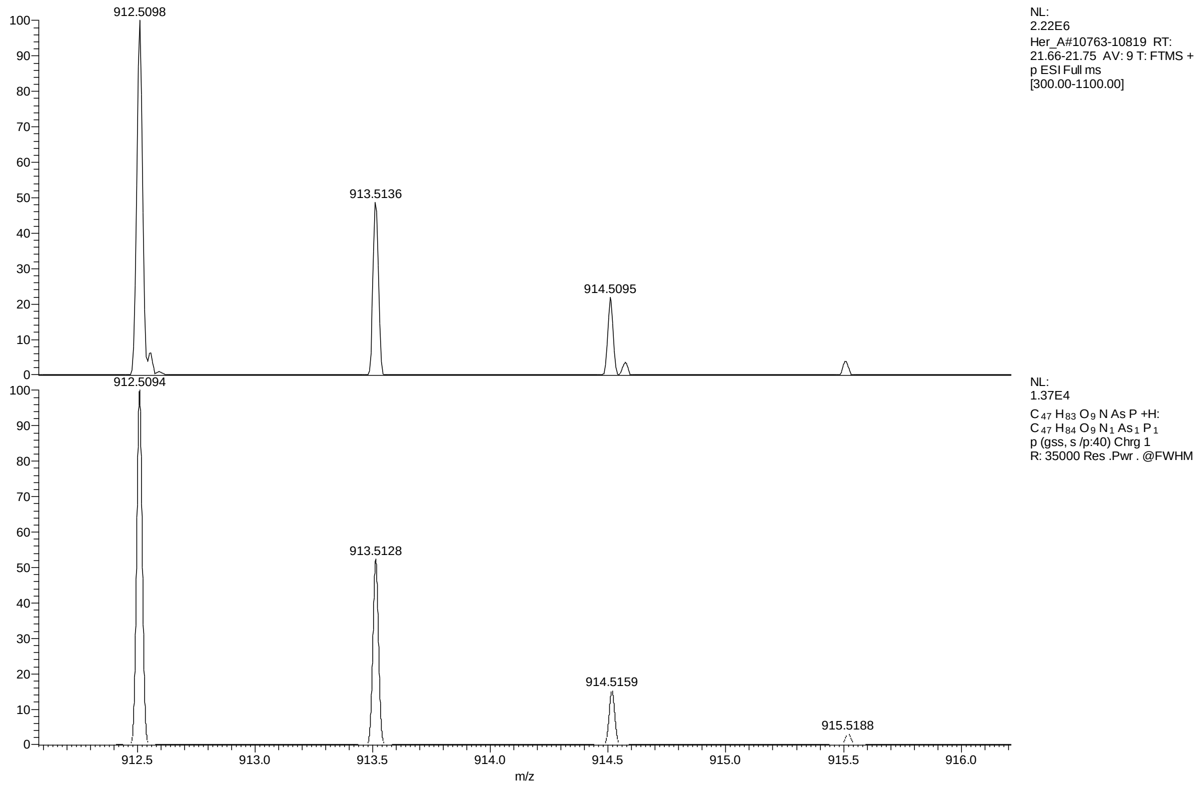

Figure S11. Measured (above) and simulated (below) isotopic pattern of AsPC 911 (singly charged, $C_{47}H_{83}O_9NAsP\_H^+$)

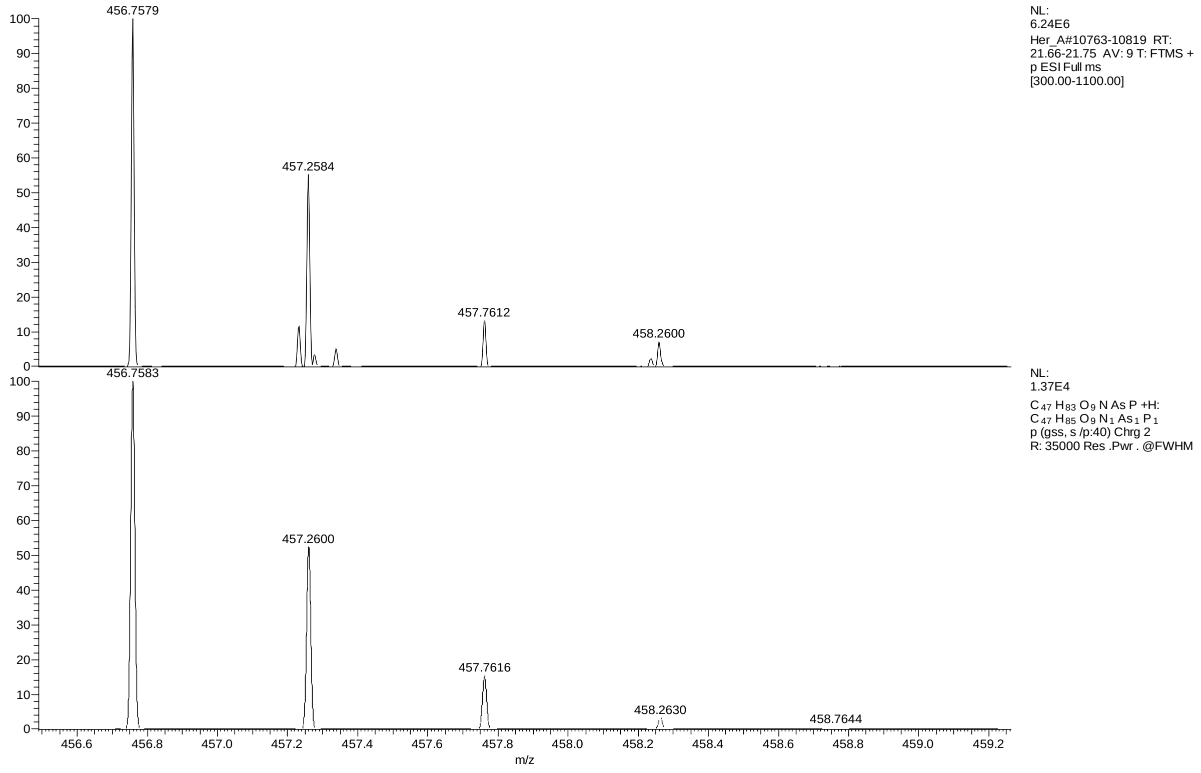

Figure S12. Measured (above) and simulated (below) isotopic pattern of AsPC 911 (doubly charged, $C_{47}H_{83}O_9NAsP\_2H^+$)

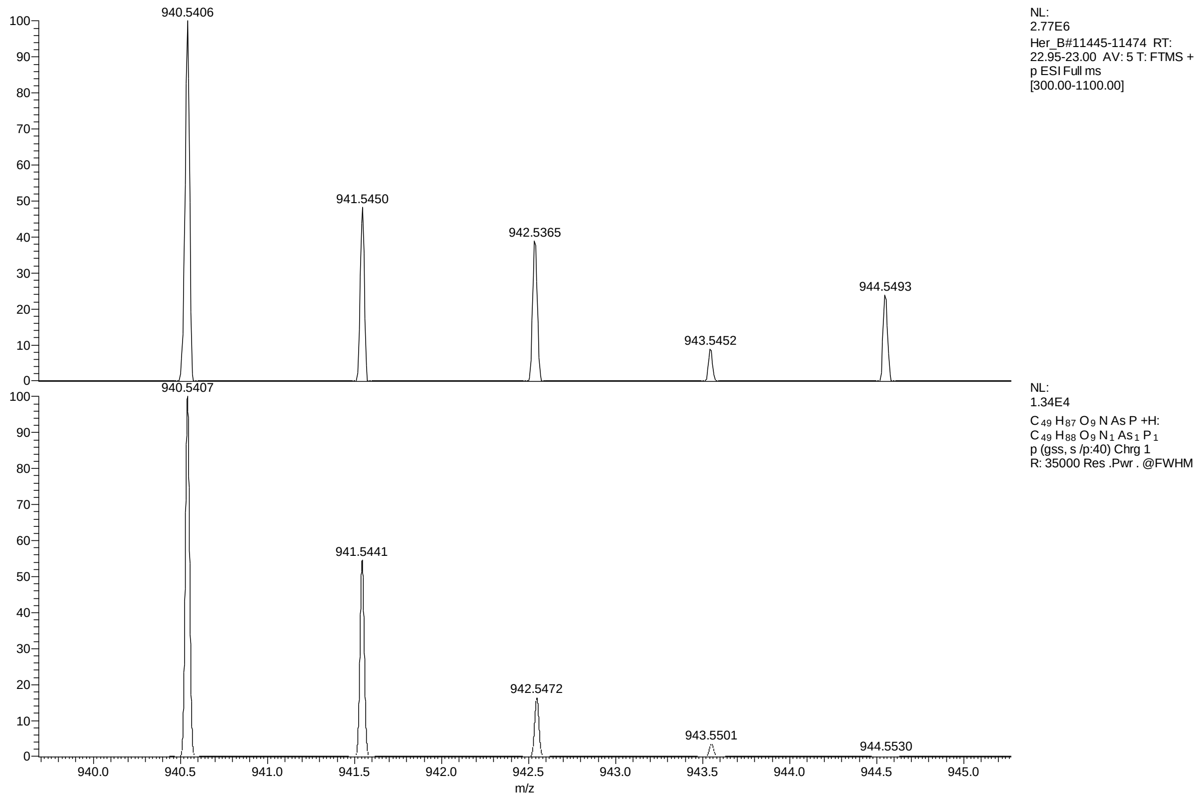

Figure S13. Measured (above) and simulated (below) isotopic pattern of AsPC 939 (singly charged, $C_{49}H_{87}O_9NAsP\_H^+$)

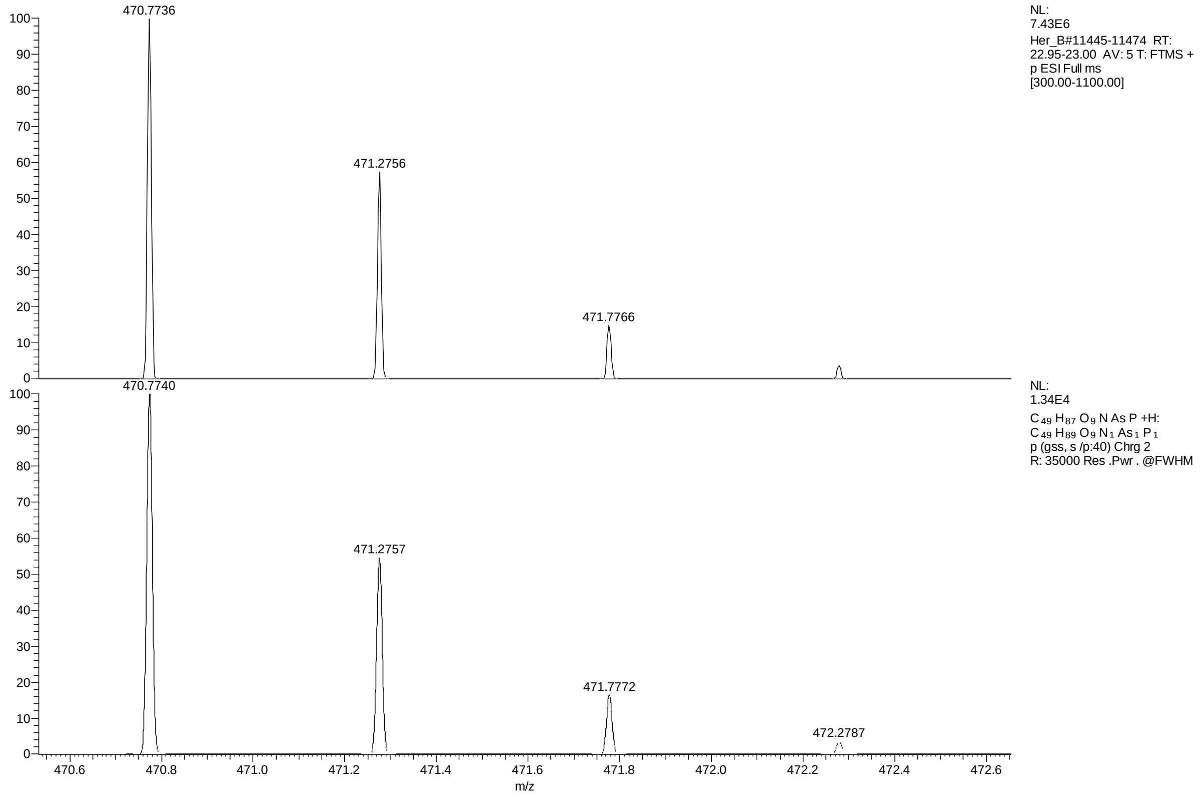

Figure S14. Measured (above) and simulated (below) isotopic pattern of AsPC 939 (doubly charged, $C_{49}H_{87}O_9NAsP\_2H^+$)

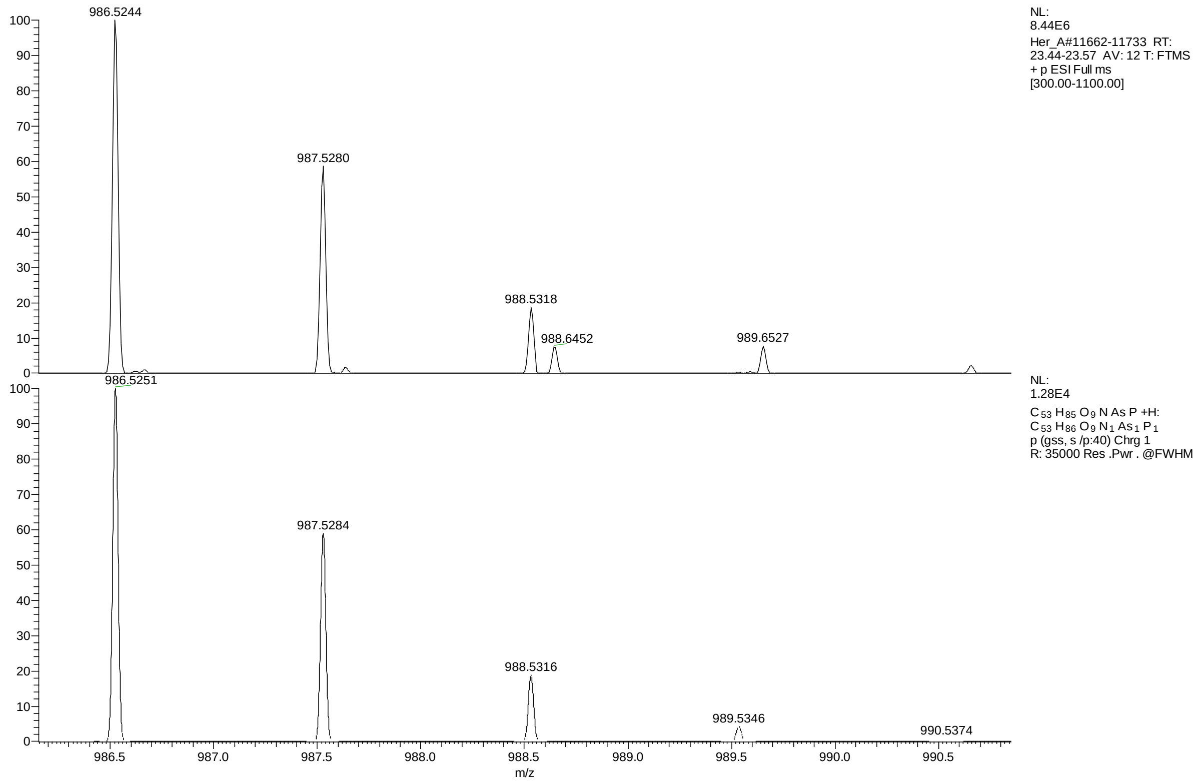

Figure S15. Measured (above) and simulated (below) isotopic pattern of AsPC 985 (singly charged, $C_{53}H_{85}O_9NAsP\_H^+$)

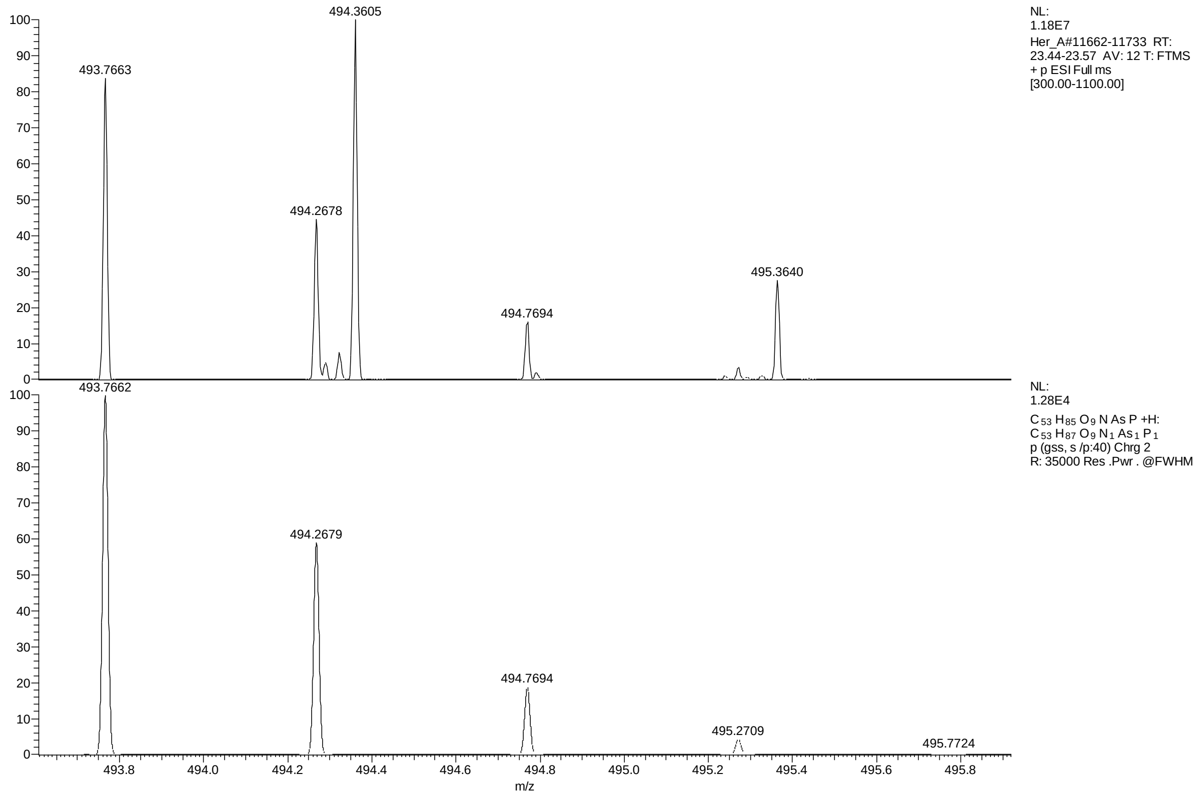

Figure S16. Measured (above) and simulated (below) isotopic pattern of AsPC 985 (doubly charged, $C_{53}H_{85}O_9NAsP\_2H^+$)

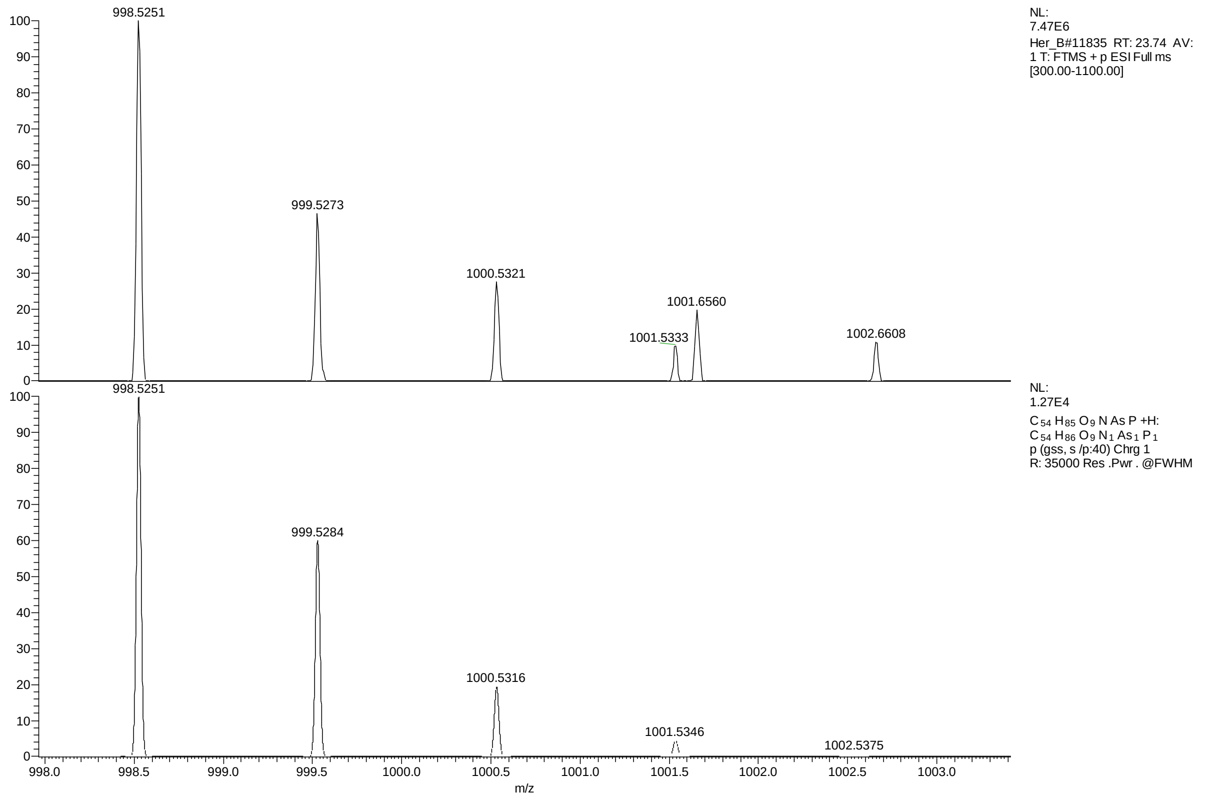

Figure S17. Measured (above) and simulated (below) isotopic pattern of AsPC 997 (singly charged, $C_{54}H_{85}O_9NAsP\_H^+$)

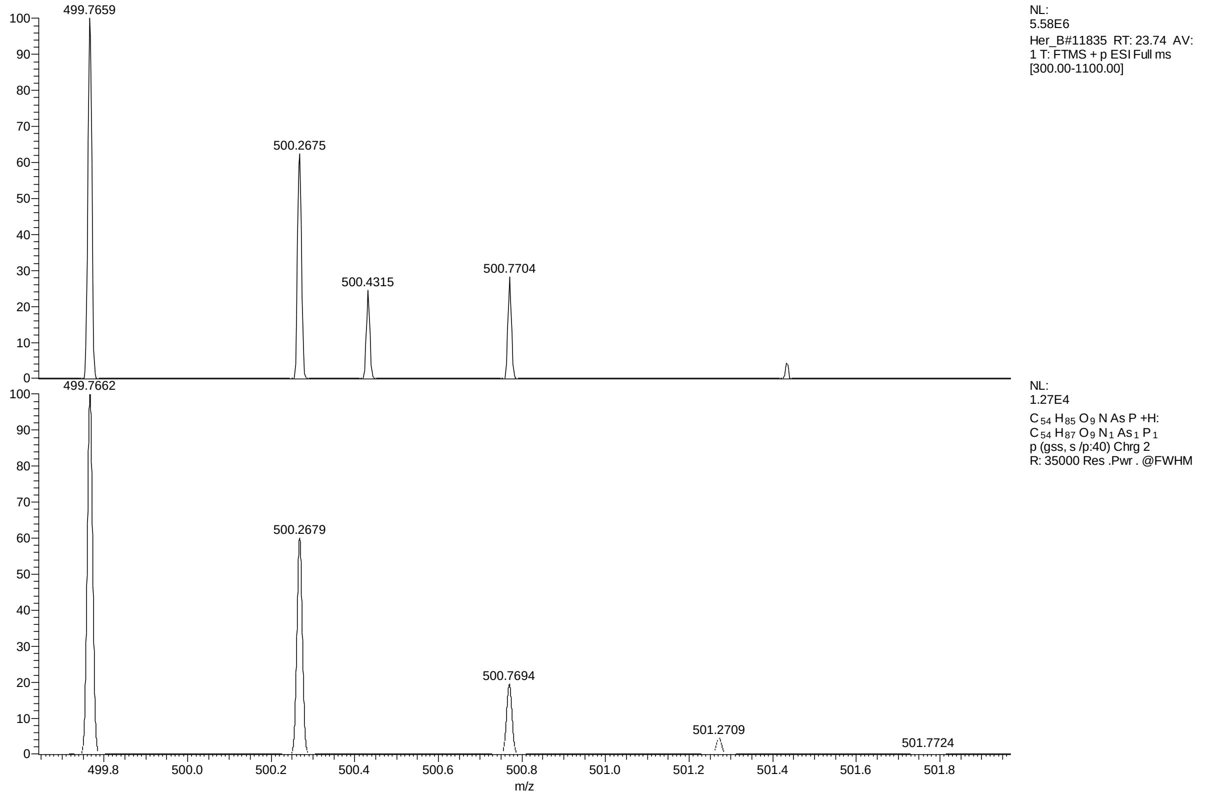

Figure S18. Measured (above) and simulated (below) isotopic pattern of AsPC 997 (doubly charged, $C_{54}H_{85}O_9NAsP\_2H^+$)

MS/MS spectra of the arsenic-containing phosphatidylethanolamine

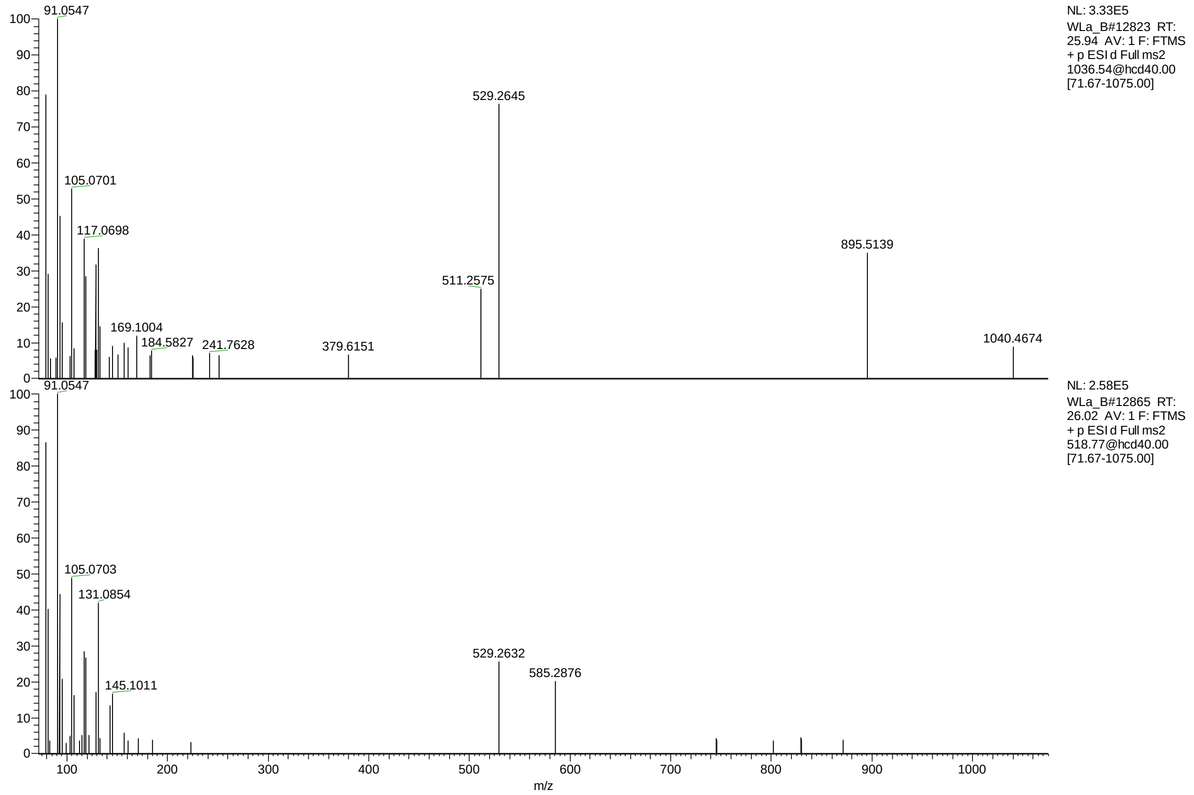

Figure S19. MS/MS spectrum of the singly charged (above, m/z=1036.5413) and doubly charged (below, m/z=518.7740) species of AsPE 1036. (neutral: $C_{57}H_{87}O_9NAsP$).

Simulated spectra of the arsenic-containing phosphatidylethanolamine

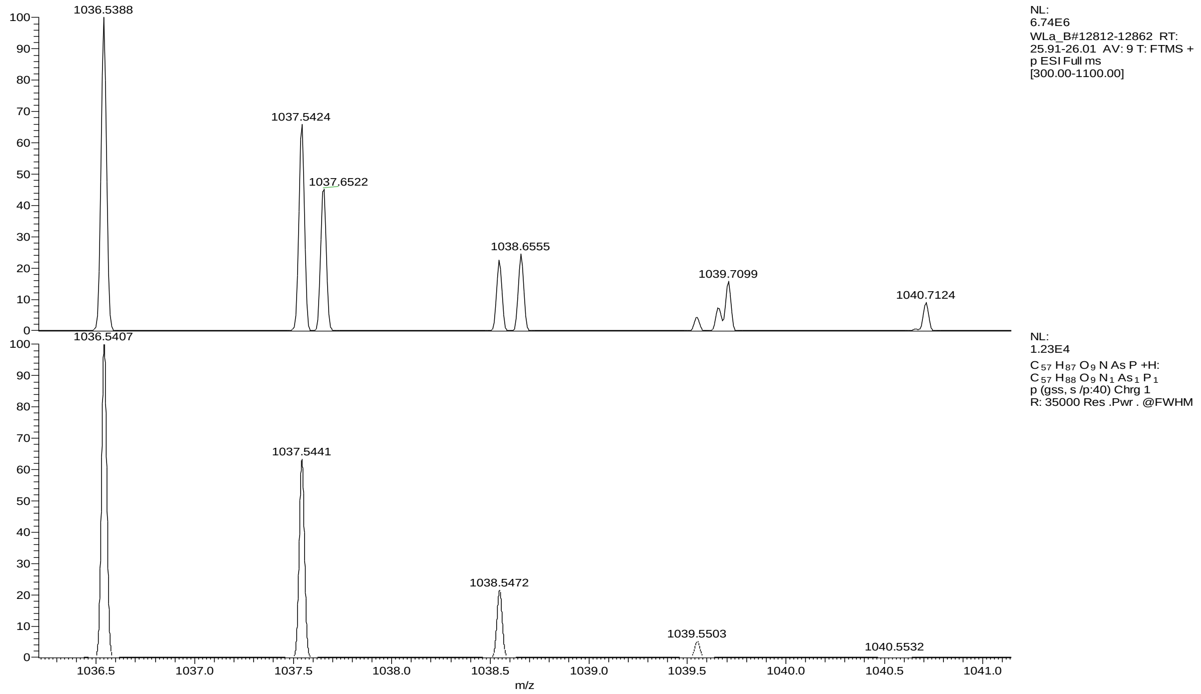

Figure S20. Measured (above) and simulated (below) isotopic pattern of AsPE 1035 (singly charged, $C_{57}H_{87}O_9NAsP\_H^+$)

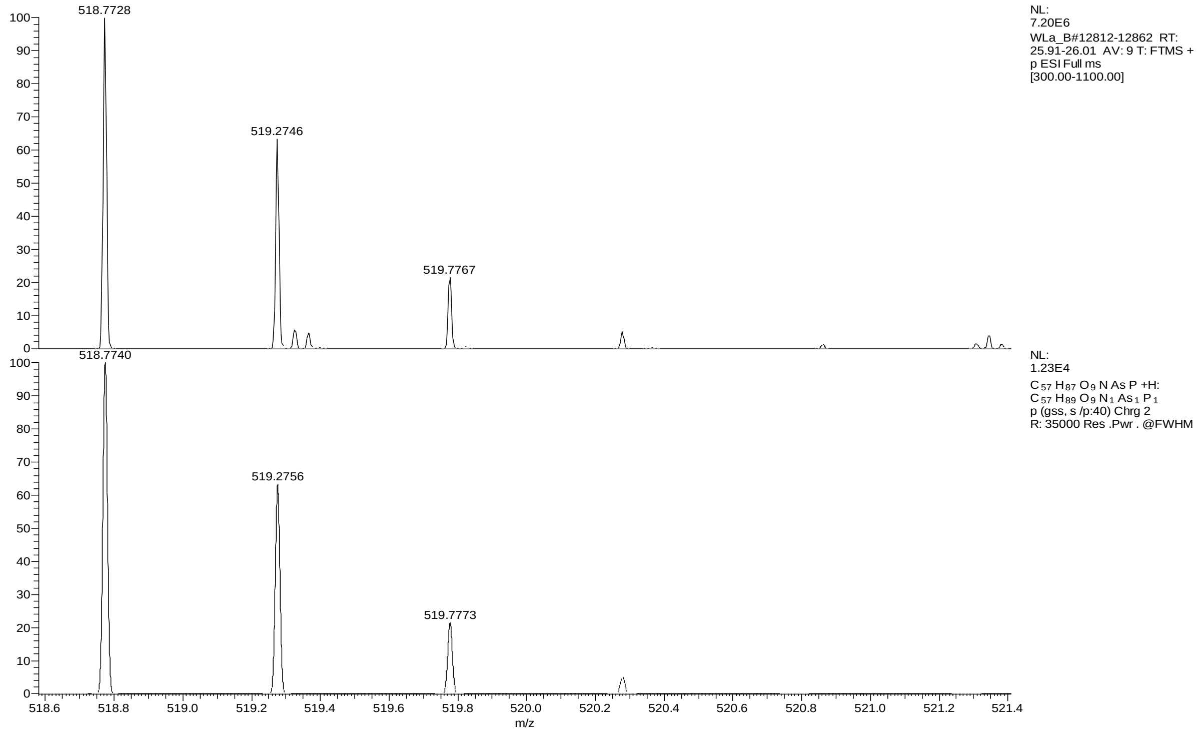

Figure S21. Measured (above) and simulated (below) isotopic pattern of AsPE 1035 (singly charged, $C_{57}H_{87}O_9NAsP\_2H^+$)

References

(1) Taleshi, M. S.; Seidler-Egdal, R. K.; Jensen, K. B.; Schwerdtle, T.; Francesconi, K. A. *Organometallics* **2014**, *33*, 1397–1403.
